# *Helicobacter pylori*-derived outer membrane vesicles: Pathogenic roles, microbiota interactions, and biomedical applications

**DOI:** 10.1016/j.jare.2025.09.055

**Published:** 2025-09-30

**Authors:** Xi Chen, Zibo Lin, Nanxi Wang, Yujie Zhou, Lei Cheng, Biao Ren

**Affiliations:** a State Key Laboratory of Oral Diseases & National Center for Stomatology & National Clinical Research Center for Oral Diseases, West China Hospital of Stomatology, Sichuan University, Chengdu, China; b Department of Operative Dentistry and Endodontics, West China Hospital of Stomatology, Sichuan University, Chengdu, China; c Hospital of Stomatology, Guangdong Provincial Key Laboratory of Stomatology, Guanghua School of Stomatology, Sun Yat‐sen University, Guangzhou, China

**Keywords:** *Helicobacter pylori*, Outer membrane vesicles, Microbiota, Virulence factors, Drug development

## Abstract

**Background:**

*Helicobacter pylori* (*Hp*) is a gram-negative, microaerophilic bacterium that infects approximate 45% of the global population. It is a key contributor to chronic gastritis, peptic ulcers, and gastric cancer. Among its secreted products, *Hp*-derived outer membrane vesicles (*Hp*-OMVs) are nanoscale proteoliposomal structures that play crucial roles in host-pathogen interactions.

**Aim of Review:**

This review aims to synthesize current knowledge on the formation, composition, and biological functions of *Hp*-OMVs, with a particular focus on their systemic effects and potential biomedical applications.

**Key Scientific Concepts of Review:**

*Hp*-OMVs are enriched with virulence-associated proteins and lipids, acting as multifunctional vehicles that facilitate the delivery of toxins, modulation of host immunity, and reshaping of gastrointestinal and oral microbial communities*.*

Emerging preclinical studies suggest that *Hp*-OMVs may translocate across epithelial barriers and reach distant organs, including the brain, where they have been shown to exacerbate neuroinflammatory responses in murine models of Alzheimer’s disease. However, no causal relationship has been demonstrated in humans. More research is urgently needed to confirm such extragastrointestinal connections.

In parallel, *Hp*-OMVs are being explored as versatile platforms for vaccine development, mucosal adjuvants, anti-adhesion therapies, and targeted drug delivery systems, owing to their intrinsic immunogenicity and tissue tropism.

**Conclusion:**

*Hp*-OMVs represent a unique vesicular modality in *Hp* pathogenesis with broad biomedical potential. Future research employing multi-omics, single-vesicle analytics, and clinical validation is needed to define their mechanistic roles and translational value.

## Highlights

• *Hp*-OMVs play a key role in *Hp* pathogenesis and virulence factor delivery.
• *Hp*-OMVs regulate gastrointestinal and oral microbiota interactions.
• Emerging links between *Hp* infection and neurological disorders via OMVs.
• *Hp*-OMVs have potential in vaccines, adjuvants, and drug delivery systems.
• Clinical applications of *Hp*-OMVs include anti-adhesion therapies and biomarkers.

## Graphical abstract

**Figure:**
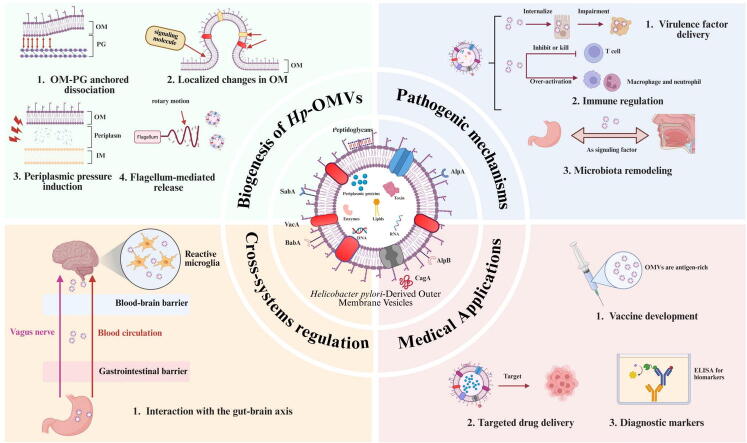

## Introduction

*Helicobacter pylori (Hp)* remains a major global public health threat despite modest declines in age‑standardized prevalence over recent decades. Current *meta*-analysis across over 100 countries indicates that almost 45 % of the global population is infected with *Hp*, and only a slight long-term downward trend is observed, with childhood prevalence persisting around one–third in many regions [1,2]. Demographic modeling shows that declining cohort‑specific prevalence is insufficient to offset population growth and rapid ageing, so the absolute number of *Hp*‑attributable diseases will remain stable or rise in several regions [3]. Gastric cancer is the fourth leading cause of cancer death worldwide, and it’s predicted that about 76 % of gastric cancer cases will be attributable to *Hp* [4]. In addition to its established involvement in gastrointestinal disorders including peptic ulcers, chronic gastritis, and gastric cancer [5], evidence shows significant associations between *Hp* infection and extraintestinal diseases such as neurological diseases, metabolic syndrome, and cardiovascular conditions, which result in substantial healthcare utilization and quality–of–life losses [[6], [7], [8]]. Nevertheless, the exact processes behind its multisystem pathogenicity have yet to be thoroughly elucidated.

*Hp*-derived outer membrane vesicles (*Hp*-OMVs) are multifunctional nanoparticles that transport lipids, virulence factors, and genetic material. These nanostructures play pivotal roles in modulating pathological processes during host colonization, particularly via immune evasion and pathogenic mechanisms [9]. *Hp*-OMVs, significant nanoscale secretory entities actively released by *Hp*, not only facilitate transmembrane delivery of virulence factors but also dynamically regulate the development and homeostatic maintenance of host immune systems. Recent investigations have highlighted the dual regulatory roles of *Hp*-OMVs in pathogen-host interactions. This review will thoroughly explain the biosynthesis processes, molecular composition, and biological functions of *Hp*-OMVs, while also examining their translational potential in precision medicine applications.

## The Basics of *Hp*-OMVs

### Biogenesis of *Hp*-OMVs

**Fig. 1:**
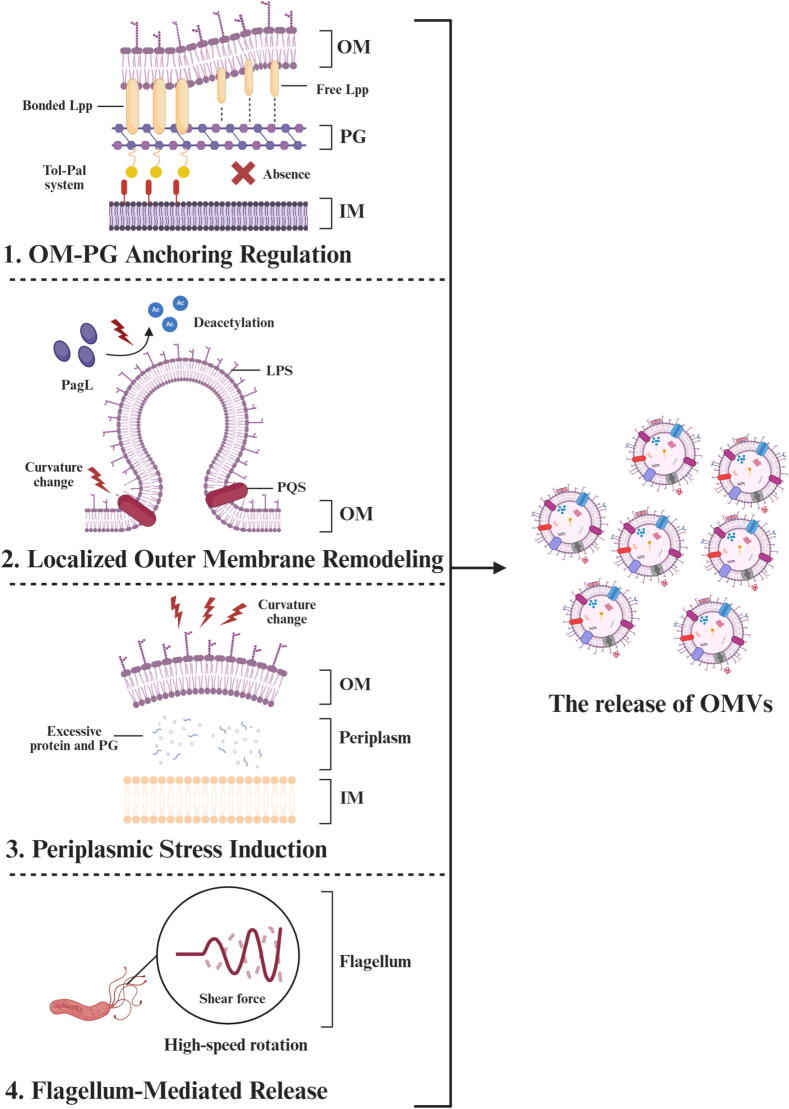
**Proposed mechanisms of OMVs production in gram-negative bacteria.** This figure summarizes four primary models of OMVs formation derived from studies in model gram-negative species, with hypothetical relevance to *Hp*-OMVs. (1) OM-PG anchoring regulation: Disruption or absence of tethering systems (e.g., Tol-Pal system) between the OM and PG reduces membrane constraint and facilitates OM protrusion and vesicle release. (2) Localized OM remodeling: Altered lipid organization, such as insertion of small molecules (e.g., PQS) or lipid A modifications (e.g., deacetylation), induces local curvature and instability in the OM which promotes vesiculation. (3) Periplasmic stress induction: Accumulation of misfolded proteins, PG fragments, or periplasmic cargo creates pressure that drives OM bulging and vesicle budding. (4) Flagellum-mediated release: Physical stress and envelope deformation caused by flagellar rotation may initiate vesicle formation. These mechanisms may operate independently or in combination to regulate OMVs release, and their relative contributions in *Hp* remain to be fully elucidated. (Created in https://BioRender.com).

Although current research specifically emphasizing that the formation of *Hp*-OMVs remains limited, understanding their formation is important for study how *Hp* regulates vesicle content, modulates host interactions, and adapts to environmental stress. To provide insight towards this gap, we summarize hypothetical models of OMVs generation in gram-negative bacteria, particularly *Escherichia coli* (*E. coli*) and *Pseudomonas aeruginosa* (*P. aeruginosa*). By exploring the commonalities in gram-negative bacteria, and combining with current researches on the formation pathway of *Hp*-OMVs, we aim to propose possible secretion approaches of *Hp*-OMVs that may guide future research on *Hp*-OMV biogenesis. (Fig. 1): (1) the outer membrane (OM)-peptidoglycan (PG) anchoring dynamics, (2) localized outer membrane restructuring, (3) periplasmic pressure induction, and (4) flagellum-mediated release.

#### OM-PG anchoring regulation

Controlled weakening of the OM-PG anchoring is a potential way of OMVs production in gram‑negative bacteria. Direct evidence in *Hp* remain comparatively limited. However, existing *Hp* mutant phenotypes together with well‑characterized paradigms from other species describe a provisional model in which focal relaxation of OM-PG permits vesicles release.

*Hp* contains two important PG metabolic enzymes, AmiA and MltD. The absence of the two enzymes leads to abnormal cellular morphology and is associated with the substantial release of chain-like *Hp*-OMVs. This observation builds a link between genotype and vesiculation, which indicates that the disintegration of the PG is related to the spatial heterogeneity of *Hp*-OMVs release [10]. Components of the Tol-Pal system, including the PG-associated lipoprotein (Pal), contribute to envelope constriction coordination. As a core part of the Tol-Pal system, Pal critically maintains inner membrane (IM)-OM connectivity. System disturbance causes OM-PG dissociation and greatly boosts *Hp*-OMVs generation [11].

Studies in several gram-negative bacteria, including *E. coli* and *P. aeruginosa*, have provided detailed insights into the role of OM–PG interactions in OMVs formation, although species-specific differences remain to be clarified. The release of OMVs entails the active dissociation of the OM-PG anchoring. The intricate composition of OM proteins in gram-negative bacteria include multiple OM-associated mediators of vesicle secretion. Specifically, the OM-localized lipoproteins Lpp (major lipoprotein) and Pal jointly maintain OM-PG interface integrity. A deficiency in these components disrupts membrane permeability, weakens covalent OM-PG linkages, and markedly enhances OMVs production [[12], [13], [14]]. PG hydrolases destabilize OM-PG interactions by disrupting its integrity. The nlpI gene in *E. coli* modulates the expression of Spr, a PG hydrolase, and mutations in this gene lead to a hypervesiculation phenotype in *E. coli* [15]. Likewise, OmpA-family proteins, which are universally found in the OM of gram-negative bacteria, exhibit conserved PG-binding domains. Their regulatory duties in OMVs formation have been mechanistically demonstrated in *Porphyromonas gingivalis* (*P. gingivalis*), *Haemophilus influenzae*, and *P. aeruginosa* [[16], [17], [18], [19]]. These paradigms frame testable analogies for *Hp* but require organism-specific validation. Although these findings are mainly based on *E. coli*, a proposed mechanism of Lpp-mediated vesiculation suggests a dynamic equilibrium between Lpp-PG binding, which bacteria regulate by coordinated PG production and degradation to promote vesicle release [13,14].

Since *Hp* lacks the classic Lpp component, it may be more dependent on the Tol-Pal system and the *Hp*-specific lipoproteins (such as HP0135) to maintain the integrity of the OM [20]. The OM adhesins of Hop, including BabA, SabA, and OipA, etc., adopt a similar β-barrel structure, interrupted by a large extracellular domain, with the N-terminus located in the periplasm [21]. These domains may have short-term low-affinity interactions within the periplasm and be involved in the connection of OM-PG, although there is currently a lack of direct data.

#### Localized Outer Membrane Remodeling

Localized perturbations of OM lipid organization create curvature that nucleate OMVs. While canonical lipid remodeling paradigms are elucidated in *Pseudomonas* and *Enterobacteriaceae*, this regulation model are still unknown in *Hp*.

The bilayer-coupling hypothesis proposes that *P. aeruginosa*-derived pseudomonas quinolone signal (PQS) is transported from the IM to integrate into the OM, where it promotes localized curvature changes in the LPS-enriched outer leaflet, thus triggering OMVs release [22]. Exogenous PQS supplementation restores OMVs production in PQS-deficient mutants while facilitating quinolone encapsulation [23]. PQS exhibits a conserved OMVs-inducing ability across Gammaproteobacteria. Furthermore, culture supernatants from *E. coli* and *Klebsiella pneumoniae* enhance *P. aeruginosa* vesiculation [24], suggesting the existence of conserved OMVs-inducing factors across bacterial species. This implies possible cross-species vesiculation modulators (e.g., quorum-sensing molecules) that could similarly influence *Hp*-OMVs biogenesis, warranting additional investigation in *Hp*.

LPS, as a core OM component, undergoes structural modifications that directly modulate OMVs biogenesis. Gentamicin enhances OMVs release by chelating divalent cations (Ca^2+^/Mg2^+^) in LPS, disrupting OM architecture [25,26]. In *salmonella*, the OM-localized enzyme PagL deacylates lipid A moieties of LPS, inducing unstable cylindrical or conical membrane configurations that facilitate vesiculation [27]. LPS variations in *P. aeruginosa* lacking lipid A or O-polysaccharide maintain normal OMVs production levels but alter vesicle size and cargo composition [28], indicating LPS modifications participate in cargo sorting during OMVs formation. These findings collectively suggest that LPS serves multiple functions in both biogenesis and cargo selection of OMVs.

For *Hp*, the lipid A in *Hp*’s LPS undergoes a series of dephosphorylation and deacylation during its formation, which is similar to the regulation of membrane stability by PagL in Salmonella [29]. This may contribute to the formation of an unstable membrane structure. In conclusion, the enzymatic regulation of LPS production and modification need thorough examination when exploring the mechanisms of *Hp*-OMVs creation. Regarding the understanding of *Hp*-OMVs in the aspect of localized OM remodeling, it is very limited. By using methods such as metabolic labeling of LPS, low-temperature electron tomography, and lipidomics, it is possible to assess the role of lipid remodeling in *Hp*-OMVs.

#### Periplasmic Stress Induction

Accumulation of misfolded proteins or PG fragments in the periplasm destroy the envelope homeostasis and can be relieved by OMVs secretion, which exports excess material and restores the balance of periplasm.

In *E. coli* and *P. aeruginosa*, loss of the periplasmic serine protease DegP or its functional homolog MucD leads to hypervesiculation because aberrant outer membrane proteins (OMPs) accumulate in the periplasm [30,31]. In Vibrio cholerae (*V. cholerae*), a small non-coding RNA (sncRNA) called vrrA downregulates the transcription of the OMP gene in a σ^E^-dependent manner to alleviate the envelope stress [32]. Additionally, the periplasmic retention of PG fragments generates localized pressure gradients that drive vesiculation, which have been already proved in *P. gingivalis* [33]. These findings collectively identify periplasmic stress dysregulation as an important initiator of OMVs release.

Although direct evidence for a periplasmic stress-induced mechanism of *Hp*-OMVs release is currently lacking, several lines of evidence may indirectly support this possibility. HtrA, the *Hp* homolog of the DegP protease, behaves both protease and chaperone activities. In htrA mutants, misfolded proteins accumulate in the periplasm, and HtrA itself has been detected embedded within *Hp*-OMVs [34]. In addition, *Hp* expresses a SurA-like protein (HP0175), which is involved in the formation of β-barrel structure. Structural mutations in such chaperones could potentially result in the misfolding or aggregation of OMPs, providing additional stress signals that may contribute to *Hp*-OMVs production [35]. Subsequent researches are warranted to determine whether disruptions in periplasmic folding or envelope stress sensing actively drive Hp-OMVs, and whether such vesiculation contributes to its persistent colonization and adaptation under gastric stress.

#### Flagellum-Mediated Release

Rotation of sheathed flagella generates local mechanical stress that can transiently disrupt the stability of junction, allowing membrane blebs to pinch off as OMVs. The idea is verified in Vibrio spp: In *Vibrio fischeri* and *V. cholerae*, sheathed flagella release LPS-riched OMVs from the flagellar base, and the shedding rate scales with flagellar density [36,37].

*Hp* shares the key anatomical prerequisites for this mechanism. *Hp*'s sheathed flagella demonstrate structural continuity with the OM, sharing LPS composition and integrated membrane proteins [38,39]. Two sheath–localized lipoproteins strengthen the indirect proves, including HP0018 and HP0135. The impairment of them could result in more production of *Hp*-OMVs [10,20]. What’s more, a newly researcher OM ring accessory called FapH safeguards membrane integrity during flagellar rotation. Its absence heightens antibiotic sensitivity and suggests that rotational stress can damage the sheath if not properly buffered [40].

In summary, these findings imply that the flagellar base in *Hp* is a credible method for OMVs biogenesis, with mechanical shear and special lipoproteins acting in concert. To convert this potential model into demonstrated mechanism, live cryo‑ET or fluorescence microscopy should be applied to track OMVs emergence in real time. Proteomics of vesicles released under different rotation would reveal whether a flagellum‑associated route contributes a distinct OMVs enriched for sheath lipoproteins and other cargoes. Such studies will explain whether the release of flagellum-related OMVs is a selective process, a random process, or a combination of both.

#### Additional Mechanisms

The VacJ/Yrb ATP-binding cassette transporter system which can be regarded as Mla pathway, evolutionarily conserved in gram-negative bacteria, preserves OM phospholipid asymmetry through selective PL transport. Targeted disruption of this mechanism has been shown to dramatically increase OMVs production in *Haemophilus influenzae*, *V. cholerae*, *E. coli*, and phylogenetically distant species [41,42].

According to the report, *Hp* has homologs of MlaD/E/F, but lacks homologs of MlaA/B/C. Although this is slightly different from the fully understood Mla pathway in *E. coli*, we propose that *Hp*-OMVs biosynthesis may be related to Mla pathway. It is worth noting that the Mla pathway is believed to be associated with the metabolism of glycerophospholipids in the OM, which confirms the mechanism of OM remodeling [40]. Evidence from various gram‑negative models with genomic conservation in *Hp* underscores the role which phospholipid asymmetry plays in *Hp*-OMVs control. Such kind of regulation maybe to some extent compatible with the regulatory mechanisms of OM-PG and OM remodeling. The next step in the research of this mechanism is to generate *Hp* bacterial strains with mla gene knocked out, and combine fluorescent probes to quantify the number of OMVs, in order to clarify the potential interactions among the above three mechanisms.

In summary, the production and release of *Hp*-OMVs likely involve multiple conserved mechanisms, including OM-PG Anchoring Regulation, localized OM remodeling, periplasmic stress induction, and flagellum-associated membrane dynamics. While direct evidence in *Hp* remains limited, studies on mutants of amiA, mltD, pal, and htrA, as well as sheath-associated proteins, could support the contribution of these pathways. These mechanisms are not mutually exclusive and may act in combination to shape vesiculation under stress or during host adaptation. Further *Hp*-specific studies, including gene deletions, lipidomics, and high-resolution imaging, are needed to verify these models and clarify their relative contributions.

### Composition of *Hp*-OMVs

**Fig. 2:**
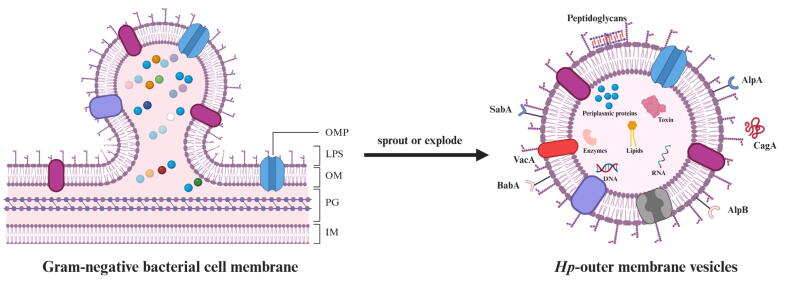
**Biogenesis and composition of OMVs.** OMVs are derived from the OM of gram-negative bacteria, and their vesicular structure is similar to the OM, containing LPS, OMP and other components. The production mode can be divided into budding or lysis. *Hp*-OMVs inherited a variety of virulence factors from the parent strain, including CagA, VacA, AlpA/B, BabA and SabA, which are important vectors for the biological effects of *Hp*. (Created in https://BioRender.com).

*Hp*-OMVs are bilayer vesicular structures ranging from 20 to 250 nm in diameter, released from the bacterial OM, and exhibit phospholipid profiles that closely resemble the parental membrane. Characteristic components include phosphatidylglycerol, phosphatidylethanolamine, and LPS [43] (Fig. 2). Compared to parental bacteria, *Hp*-OMVs selectively accumulate virulence factors such as CagA and VacA, as well as enzymatic constituents including catalase KatA, serine protease HtrA, and γ-glutamyl transpeptidase [9,44,45].

Under iron-limited conditions, *Hp*-OMVs undergo Lewis Y antigenic epitope modification on LPS, accompanied by a reduction in VacA and an increase in proteases, indicating a stress-responsive compositional adaptation [46,47]. It is hypothesized that based on conserved patterns in gram-negative bacteria, that *Hp* achieves maximal OMVs secretion during the logarithmic growth phase, although species-specific regulatory mechanisms require experimental validation [48]. We hypothesize that *Hp* uses its own set of regulatory systems to control molecules which are packed into OMVs, depending on both external conditions like iron availability and its own growth stage. To clarify these mechanisms, more detailed studies using synchronized cultures and metal-controlled environments are needed, especially with genetically similar bacterial strains and advanced protein analysis tools.

Imbalanced lipid distribution promotes OMVs budding in many gram-negative bacteria, and *Hp* also follows this trend. Lipidomics reveals that *Hp*-OMVs are enriched in curvature-inducing glycerophospholipids, especially lyso-phosphatidylcholine (LPC) 15:0 and LPC 18:0, rather than simply replicating the OM composition. These LC-MS/MS-identified components are related in glycerophospholipid metabolism and autophagy pathways [49]. In contrast, the bulk Hp membrane contains almost 70 % phospholipids and up to 25 % uniquecholesterol glucosides, whose rigidity and order likely restrain vesiculation [50].

In light of this, the compositional landscape of *Hp*-OMVs merits systematic overview to clarify its potential biological functions. Table 1 summarizes the currently reported components of *Hp*-OMVs based on proteomic, lipidomic and transcriptomic analyses.

**Table 1:** Characterized molecular components of *Hp*-OMVs.

**Components**	**Functions**	**Reference**
VacA	Induction of vacuolation; Immunosuppression	[51]
CagA	Promote inflammation; carcinogenesis	[52]
UreA/B	Neutralize acid	[49,51]
BabA; SabA; OipA; Hop; AlpA/B	Adhering to the gastric epithelium; Mediating receptor binding	[45]
HtrA	Disrupting cell connections	[34]
KatA	Neutralizing oxidizing substances	[44]
Hcp; MtrC	Hydrolyzed antibiotics	[53]
PG	Activate innate immunity	[54]
PGly; PE; LPE; PC; LPC; CL; LPS	Immune regulation; Potential pathogenic effect	[45,49]
ChG; CAG	Enhancing adhesion and CagA transport	[55]
*Hp*-deprived DNA and RNA	Horizontal gene transfer; Transcriptional regulation	[56]

**Abbreviations**.

PG, peptidoglycan; PGly, phosphatidylglycerol; PE, phosphatidylethanolamine; LPE, lyso-phosphatidylethanolamine; PC, phosphatidylcholine; LPC, lyso–phosphatidylcholine; CL, cardiolipin; LPS, lipopolysaccharide; ChG, cholesteryl α–D–glucopyranoside.

### Techniques for OMVs Isolation and Characterization

The yield, purity and analysis of OMVs largely depend on methods used for their separation and characterization. Currently, several techniques have been developed, each with its own advantages and limitations. When comparing experimental results or determining clinical applicability, these factors should be carefully considered.

#### Isolation and Enrichment

Ultracentrifugation (UC) remains the most commonly used technique for OMVs separation and enrichment. Differential ultracentrifugation enables batch precipitation of vesicles based on sedimentation rate, while density gradient ultracentrifugation separates vesicles according to buoyant density to enhance purity. However, both of these methods are time-consuming and may result in changes in vesicle morphology or contamination by proteins. Compared with UC, ultrafiltration behaves a higher recovery rate and less interference, but has a lower recovery rate for larger vesicles [57,58]. Size exclusion chromatography (SEC) is a more gentle, scalable alternative. SEC can effectively remove soluble proteins and achieve simple and efficient purification of OMVs, demonstrating good consistency and reproducibility. The precipitation technique is usually based on polyethylene glycol, which is rapid and high-yielding, but the products are more prone to contamination and is not suitable for analyses requiring precise detection [59,60]. In addition, there are some emerging separation methods. For instance, the antibody-based specific enrichment method for OMVs. By targeting the characteristic molecules of *Hp* (such as VacA or CagA), it is possible to selectively enrich *Hp*-OMVs under complex background conditions. But the high costs and the potential risks of omissions still need to be overcome [61]. The method based on microfluidic technology has the characteristics of fast speed, high efficiency and high sensitivity in the separation of OMVs. However, the research on this technology in the field of detection is still in its infancy. Specialized equipment and unified detection standards are lacking [62,63].

#### Characterization and Quantification

Transmission electron microscopy, cryogenic electron microscopy, and atomic force microscopy can be used to directly observe OMVs. But the technical sensitivity and high cost of sample preparation have limited their wide application. Nanoparticle tracking analysis (NTA) and tunable resistance pulse sensing (TRPS) are widely used for the analysis of particle size and concentration. NTA offers real-time particle tracking and is compatible with fluorescent labeling, but it is also susceptible to background particles. TRPS provides higher particle size measurement accuracy, but is more labor-intensive and delicate to blockage [64]. Single-particle phenotypic analysis techniques, such as nanoparticle flow cytometry and interferometric reflection imaging sensors, place a new option for characterizing the size and surface markers of OMVs. The limitations of this type of technology lie in the lack of a reasonable marking scheme and high costs [65]. Considering that the OMVs carry bacterial-specific contents, by using methods such as proteomics, lipidomics and RNA profiling, not only can the composition of OMVs be fully analyzed, but also background contamination can be excluded [66].

In conclusion, with the advancement of methodology and the increasing emphasis on the standardization of OMVs, we advocate the use of multiple different methods in research to enhance the comparability and reproducibility of the results. Various advanced methods need to be applied in *Hp*-OMVs.

## Pathogenesis of *Hp*-OMVs

### Virulence Factor Delivery

OMV exemplifies a distinct pathogenic mechanism by which virulence factors are transmitted to host cells through various pathways. The biological properties of OMV-encapsulated virulence factors may be altered during this process. *Hp*-OMVs exert pathogenic effects via multiple internalization pathways, with their size characteristics playing a crucial role in determining the selection of these pathways.

*Hp*-OMVs of different sizes transport virulence factors including CagA and VacA, entering host cells through three main mechanisms: macropinocytosis, clathrin-dependent endocytosis, and caveolin-mediated endocytosis, with smaller *Hp*-OMVs showing a preference for caveolin-mediated pathways [67]. The internalization efficiency of *Hp*-OMVs is significantly influenced by the cholesterol levels and membrane fluidity of host cells [68]. This size-internalization mechanism correlation may influence the tissue targeting specificity and pathogenic effects of *Hp*-OMVs within the host.

*Hp*-OMVs deliver virulence factors (VacA, CagA, UreA) to host cells via mechanisms that are dependent on time and dosage [51]. Both soluble VacA and *Hp*-OMVs-bound VacA are internalized by gastrointestinal epithelial cells, leading to cytotoxic effects. For instance, *Hp*-OMVs-treated AGS cells exhibit glutathione depletion, formation of destructive chromosomal micronuclei, and a marked reduction in proliferation [69,70]. Notably, this OMVs-associated VacA comprises 25 % of total VacA and demonstrates enhanced immunomodulatory activity compared to free forms, especially in the regulation of mucosal immunity [71].

*Hp*-OMVs deliver a coordinated set of virulence cargos that converge on host epithelial hallmarks, including junctional integrity, polarity, inflammatory amplification, membrane microdomain organization, and autophagy. Thereby fostering a pro‑carcinogenic mucosal milieu. *Hp*-OMV‑associated CagA selectively targets at ZO‑1-enriched tight junctions (TJs), perturbing junctional organization, while UreA delivered by *Hp*-OMV gains nuclear targeting capacity that exacerbates epithelial polarity loss and may potentiate the CagA-driven “hummingbird” cytoskeletal phenotype [[72], [73], [74]]. In parallel, such kind of combined CagA and LPS exposure elevates reactive oxygen level and activates NF–κB, upregulating IL–6 and TNF–α. This inflammatory signaling suppresses epithelial proliferative renewal, enhances apoptosis, and contributes to endothelial injury, which contributes to the formation of chronic inflammatory niche [73]. Vesicular cholesteryl α‑D‑glucopyranoside acyltransferase (CGAT) promotes in situ synthesis of cholesteryl 6′‑O‑acyl‑α‑D‑glucopyranoside (CAG) to stabilize lipid rafts, which reinforces bacterial adhesion, and builds a platform for the engagement of downstream virulence factor [55]. Moreover, co–internalized PG fragments activate cytosolic NOD1, driving NF–κB dependent IL–8 secretion which leads to recruitment of inflammatory cell, and simultaneously inducing RIP2–dependent autophagy that functionally intersects with VacA–modulated autophagy [75].

To sum up, Hp-OMVs serve as protective carriers for virulence factors, which enable targeted subcellular localization, establish both temporal and spatial conditions for their non-canonical biological activities.

### Gastric Mucosal Injury and Carcinogenesis

Adhesion is essential for *Hp*-OMVs-mediated gastropathy. Proteomic investigations have identified that *Hp*-OMVs comprise many adhesion-related components, including the adhesins AlpA and AlpB. These surface-exposed proteins are invariably seen in *Hp*-OMVs, with AlpA primarily mediating gastric epithelial adhesion, whilst AlpB further aids in biofilm formation [76]. VacA additionally promotes the binding of *Hp*-OMVs to host cells via interactions with lipid rafts [77]. In particular, non-acidic environments boost the uptake of *Hp*-OMVs. This pH-dependent internalization pattern suggests that *Hp*'s urease-mediated acid neutralization not only helps bacterial colonization but also improves the effectiveness of virulence factor delivery, therefore expediting the progression of *Hp*-related mucosal injury [78].

*Hp*-OMVs generally mediate gastric mucosal pathogenesis with immunological dysregulation and modulation of oncogenic signaling. *Hp*-OMVs induce apoptosis in AGS cells by means of a VacA-independent mechanism that activates caspase-3 and caspase-8, concurrently initiating mitochondrial-independent autophagy to cause gastric epithelial damage [79]. These vesicles also regulate p53 signaling via downstream effectors AIFM2 and IGFBP3, activating serine/threonine kinase 2 signaling to drive autophagic flux and amplify gastric inflammatory responses [80]. Eosinophil-*Hp*-OMVs interactions, whether through direct contact or paracrine signaling, producing eosinophil cationic protein that directly harms gastric epithelium and attracts inflammatory infiltrates [81].

Although *Hp*-OMVs have been shown to activate multiple carcinogenesis pathways including NF-κB, APK/ERK and OSM/OSMR. These observations primarily arise from in vitro gastric epithelial cell models or speculation in reviews, and the extent to which each pathway contributes in vivo remains unclear. The actual effects and interactions of these pathways lack explanations through animal experiments or clinical trials. Therefore, while these pathways suggest a possible mechanism for *Hp*-OMVs-mediated epithelial inflammation and proliferative dysregulation, further in vivo studies are required to establish their relative importance in gastric carcinogenesis [[82], [83], [84]].

### Immune Evasion and Drug Resistance

OMVs mediate immune evasion with multiple immunosuppressive mechanisms. Modification of surface antigens is a critical strategy, as structural changes in vesicular components diminish immunogenicity. In the initial stages of infection, the low immunogenicity of *Hp*’s LPS allows *Hp*-OMVs to aviod effective immune recognition [85]. These vesicles orchestrate immunosuppression by two synergistic pathways: 1. Immune cell dysregulation: *Hp* infection will activate the COX-2/PGE2 pathway in gastric epithelial cells, increasing PGE2 levels. This signaling both mediates immune evasion, through PGE2-induced upregulation of IL-10/Treg cells and inhibition of DC function; and also shapes the tumor-promoting microenvironment in the context of chronic inflammation, promoting the occurrence and progression of gastric cancer. *Hp*-OMVs have been reported to drive peripheral monocytes to overexpress COX‑2, with consequent surges in PGE2 and IL‑10, which together blunt T‑cell proliferation [43,[86], [87], [88]]. By activating the Akt-mTOR-IKK-NF-κB and Akt-Nrf2 signaling pathways, *Hp*-OMVs upregulate heme oxygenase-1 in dendritic cells (DCs). This molecular cascade inhibits DCs maturation, preserving an immature phenotype characterized by reduced responsiveness to LPS stimulation [89]. 2. Immune camouflage: The LPS in *Hp* OM undergoes modifications such as phosphorylation and deacylation to form a less toxic lipid A structure, significantly weakening the recognition by TLR4 and conferring resistance to cationic antimicrobial peptides. Therefore, we believe that *Hp*-OMVs, which are derived from the OM, also exhibit this characteristic and help *Hp* evade the attack of the innate immune response [90]. Additionally, the unique CAG of *Hp* not only impair phagocytosis but also alter the organization of host immune receptors by aggregating membrane lipid rafts. The key enzyme CGAT, localized to the bacterial OM, is delivered into host cells via *Hp*-OMVs, where it synthesizes long-chain CAG locally. This process promotes raft aggregation and strengthens adhesion signaling, thereby indirectly hindering immune clearance [55,91].

An expanding repertoire of immunoregulatory constituents has been identified within *Hp*-OMVs. *Hp*-OMVs carry vesicle-encapsulated sncRNAs that enter gastric epithelial cells, thereby effectively suppressing IL-8 secretion to attenuate local immune responses [92]. The RNA-mediated immunomodulatory exhibits evolutionary conservation, as shown by *P. aeruginosa* OMVs delivering sRNAs that target host mRNAs, dampening pulmonary LPS responses and monocyte/macrophage innate immunity in murine models [93,94]. Moreover, the O-polysaccharide component of *Hp* LPS shares structural homology with human blood group antigens. Prolonged exposure to this molecular mimic may lead to the production of autoantibody via epitope spreading [45]. The similar antigenic structure in *Hp*-OMVs suggests that these vesicles may play a role in *Hp*-related autoimmune pathologies. Mechanistic studies reveal that continuous Lewis antigens exposure during chronic infection triggers gastric mucosa-targeting autoantibodies, which accelerates the progression of atrophic gastritis [85], thus offering experimental evidence for this pathophysiological connection. Therefore, we speculate that the cargos transported by *Hp*-OMVs such as lipids and RNA is involved in the immune regulation of *Hp*, and enzymes participated in lipid metabolism should also be given attention.

*Hp*-OMVs also strengthen bacterial adaptability to environmental by modulating biofilm formation. These vesicles perform as primary reservoirs of extracellular DNA (eDNA), a vital component of biofilms that modifies biofilm architecture and improves structural integrity while transmitting antibiotic resistance determinants [95]. In multidrug-resistant *Hp* strains, *Hp*-OMVs enriched with eDNA and biofilm-associated proteins significantly enhance biofilm stability. Notably, OMVs from the TK1402 strain horizontally transfer biofilm-forming capacity among bacterial communities. The nuclease-resistant properties may at the same time safeguard immunomodulatory sncRNAs from degradation, thereby promoting immune evasion [92,[96], [97], [98]]. The capacity for horizontal transfer demonstrates phylogenetic conservation, as exemplified by *E. coli* OMVs transferring nitrite reductase DNA to improve survival under anaerobic conditions [99]. Furthermore, *Hp*-OMVs neutralize bactericidal agents such as H_2_O_2_ and levofloxacin, and they counteract the activity of the antimicrobial peptide LL-37 in a dose-dependent manner. The protective effect mainly arises from direct binding between vesicles and antibiotics, enabled by interactions that depend on the lipophilicity of the antibiotics [100,101].

*Hp*-OMVs orchestrate bacterial immune evasion across coordinated molecular mechanisms, allowing persistent *Hp* colonization at gastric niches. These vesicles sustain the release of cytotoxic components that induce chronic tissue damage. Accordingly, the evidence supports the view that *Hp*-OMVs are key mediators of chronic inflammation.

## Roles of *Hp*-OMVs in the Gastrointestinal Microbiota

**Fig. 3:**
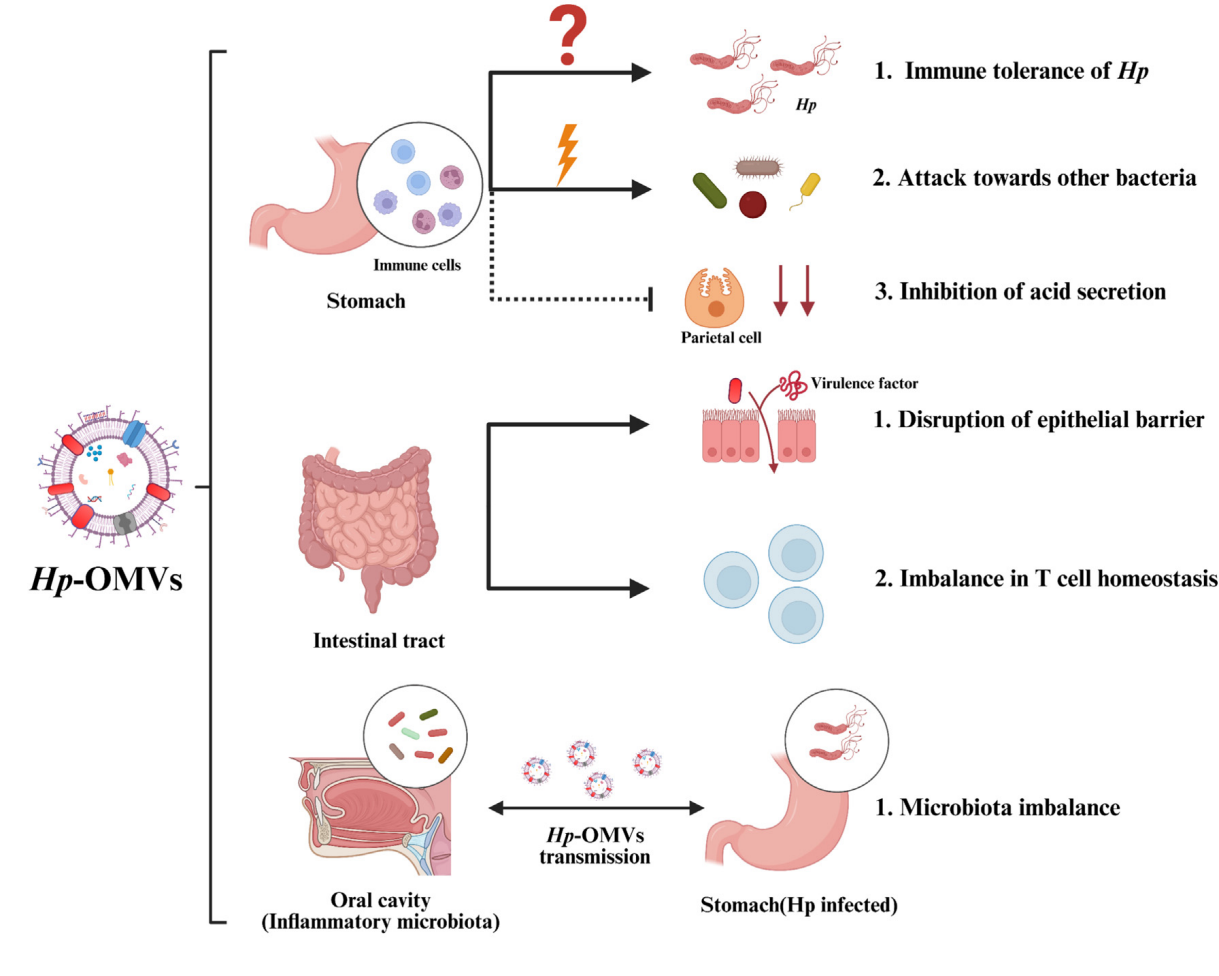
**Roles played by *Hp*-OMVs in the stomach, intestine, and oral cavity.***Hp*-OMVs assist *Hp* to acquire immune tolerance in the stomach, inhibit the survival of other microorganisms by inducing immune response, and reduce gastric acid secretion through damaging parietal cells. In the intestinal tract, *Hp*-OMVs deliver virulence factors that disrupt the epithelial barrier and disrupt CD4^+^ T-cell homeostasis. Oral cavity is a reservoir of *Hp*. Through *HP*-OMVs, *Hp* in the stomach and oral cavity may communicate with each other. (Created in https://BioRender.com).

The biological effects of *Hp* are not limited to the stomach; the downstream gut and the distal oral cavity are all associated with *Hp*. As a signal carrier, *HP*-OMVs may assist in the microbial communication of *Hp* in various sites (Fig. 3).

### Impact of *Hp*-OMVs on Gastric Microbiota

*Hp*-OMVs influence the host immune system by secreting virulence factors, which promote *Hp*-specific immune tolerance and activate antimicrobial responses against competing microbiota, including the stimulation of LL-37 production. These vesicles reduce gastric acid secretion and degrade the mucus layer, resulting in an increased intragastric pH that encourages the colonization of acid-sensitive microorganisms [102]. The trimeric HtrA protease secreted by *Hp* exhibits potent to disrupt gastric barriers, which potentially enables microbial invasion into the lamina propria [103]. However, the proteolytic activity of HtrA within *Hp*-OMVs remains uncharacterized, leaving its potential contribution to *Hp*-OMVs-mediated pathogenesis subject to further investigation. Through these mechanisms, *Hp*-OMVs promote *Hp* dominance while diminishing gastric microbial diversity and evenness. The ecological shift is characterized by increased Proteobacteria abundance, alongside decreases in the phyla Actinobacteria, Bacteroidetes, and Firmicutes [104].

### Impact of *Hp*-OMVs on Distal Microbiota

The anatomical continuity between gastric and intestinal compartments allows for the effects of *Hp* infection to extend to intestinal microbiota populations. Experimental evidence from murine fecal analyses shows that *Hp*-induced microbial alterations translocate from gastric niches to distal cecal and ileal regions [105]. *Hp*-OMVs may serve as potential long-distance signaling vectors, facilitating inter-compartmental communication in this process [106].

*Hp*-OMVs may translocate to the small intestine either directly or via the bloodstream after secretion, where virulence factors (VacA, CagA, LPS) compromise intestinal TJs, causing barrier dysfunction (leaky gut) and thereby promoting pathobiont colonization [72,107]. Concurrent suppression of gastric acid by *Hp*-OMVs potentially enables the translocation of gastric microbiota to distal regions of the gut [104]. *Hp*-positive individuals exhibit greater gut microbial diversity compared to those with gastric microbial depletion. Beyond pH modulation, *Hp*-OMVs may alter CD4 + T cell homeostasis in gut-associated lymphoid tissue through IL-10-mediated pathways, thus further promoting acid-sensitive microbial colonization [108]. The dual immunomodulatory and acid-suppressive properties may explain the inverse epidemiological relationship between *Hp* infection and the pathogenesis of inflammatory bowel disease (IBD), with *Hp*-OMVs playing indispensable roles [109].

Other OMVs of intestinal microorganisms also exhibit similar effects, suggesting that the biological functions mediated by these OMVs are likely a conserved strategy. The OMVs of *Bacteroides fragilis*, a type of intestinal probiotic, loaded with polysaccharide A act towards the TLR2 of DCs, driving the immune regulation mediated by IL-10, and preventing mice from developing colitis [110]. In contrast, the OMVs of *Bacteroides multiformis* have sulfatase activity and target macrophages, causing intensive colitis in mice [111]. Study in vitro have shown that the OMVs of adherent-invasive *E. coli* upregulate TLRs and alter junction proteins, exacerbating the imbalance of the microbial community [112]. Similarly, *Fusobacterium nucleatum*, a pathogen from the mouth, OMVs activate TLR4/NF-κB and promote intestinal inflammation in humanized microbiota mice [113].

Such an interaction between the oral cavity and the intestinal tract is also observed in *Hp*. The oral cavity and gastrointestinal tract represent highly complex microbial habitats. Current research indicates that the interaction of *Hp* with the oral-intestinal floral axis could have an impact on its colonization, infection, and pathogenicity [114]. The discovery of *Hp* in the oral cavity underscores the bidirectional interactions between oral and gastrointestinal microbiota, potentially organized by *Hp*-OMVs. As the first gastrointestinal barrier, the oral cavity acts as an extragastric reservoir for *Hp*, supporting microbial translocation between oral and gastric environments. Gastric eradication therapy modifies the composition of oral microbiota, whereas oral *Hp*, under the protection of fungi (e.g., Candida spp.), promotes gastric recolonization [115,116]. A previous study reported reduced microbial richness and diversity in oral cavity and fecal samples from patients with gastric cancer [117]. A separate study indicated that successful *Hp* eradication benefits the presence of advantageous oral microbiota, such as Lactobacillaceae and Streptococcaceae. In contrast, failure to eradicate is associated with an inflammatory microbiota, including Weeksellaceae, Neisseriaceae, and Peptostreptococcaceae [118]. Considering the competitive or symbiotic interactions between *Hp* and oral microbiota [114], we propose that *Hp*-OMVs might operate as essential signaling molecules within the oral-gastrointestinal axis. Gastrointestinal-derived *Hp*-OMVs may foster the proliferation of oral pathogens and create an inflammatory oral microenvironment, thereby supporting *Hp* survival and developing the oral cavity a hidden reservoir for recurrent *Hp* infections.

## Interaction of *Hp*-OMVs with Gut-Brain Axis

The gut-brain axis is a sophisticated bidirectional regulatory system that maintains a dynamic equilibrium between the central nervous system (CNS) and gastrointestinal functions through different pathways, including vagal signaling, endocrine mechanisms, immune responses, and gut microbial metabolites [119]. Signaling in the gut-brain axis encompasses various biological barriers, with the intestinal barrier and blood–brain barrier being especially significant.

The intestinal barrier acts as the primary defense mechanism in host-microbe interactions within the gut; besides the superficial mucus barrier, the epithelial, and vascular endothelial barriers being its most essential components. The former regulates permeability through TJs, which are composed of claudin family proteins and occluding. In contrast, the latter forms a dual-sealing structure utilizing TJs (including occludin, ZO-1, cingulin, and JAM-A) and adherens junctions (AJs, which consist of VE-cadherin/β-catenin complex). The blood–brain barrier displays more specialized structural features; however, its fundamental barrier architecture is similarly composed of TJs and AJs [[120], [121], [122], [123]]. The two barriers share clear structural homology and show closely related functional interactions. During intestinal inflammation, the choroid plexus vascular barrier can respond to gut-derived LPS signals by remodeling its TJs to enhance barrier integrity [124]. This structural–functional correspondence may provide a potential anatomical foundation for gut-brain comorbidity mechanisms mediated by microbial products, especially *Hp*-OMVs.

The ability of *Hp*-OMVs to traverse biological barriers grants them the potential to induce neurological damage. Based on in vivo studies in mice, after injection of *Hp*-OMVs, mice developed Alzheimer's disease neuropathology, which were characterized by amyloid-beta (Aβ) plaque accumulation in the brain. These plaques were found to colocalize with the injected OMVs. Besides, the extent of Aβ deposition positively correlated with the severity of cognitive impairment observed in behavioral assessments. In vitro experiments further explained that OMVs could bind Aβ peptides on their surface, thereby accelerating plaque aggregation and significantly potentiating Aβ-induced neurotoxicity. Among those components, lipid species especially LPC 18:0 appeared to mediate this process, likely by interfering with the ability of microglia to clear pathological proteins [49,125]. Based on both in vitro and in vivo experiments, we hypothesize that *Hp*-OMVs promote the progression of such neuro-pathological manifestations through the C3-C3aR signaling axis and the NF-κB pathway-induced chronic inflammation [126]. However, most evidence remains limited to in vitro and animal studies, and causal relationships have not been fully proved, and there are still few studies on *Hp*-OMVs. Further investigations are required to determine whether *Hp*-OMVs serve as core triggers of neurodegenerative processes in humans and whether they represent viable targets for therapeutic intervention.

The hypothalamic–pituitary–adrenal (HPA) axis behaves as the main humoral efferent pathway for the CNS within the gut-brain axis, integrating gastrointestinal signals to release glucocorticoids, which modulates intestinal immune function, motility, and microbiota homeostasis [127]. Corticotropin-releasing factor (CRF) functions as a key initiator of the HPA axis and is capable of bidirectionally regulating intestinal inflammation and permeability [128,129]. Research indicates that the *Hp* virulence factor VacA starts anorexia and anxiety-like behaviors by activating the Ucn1-CRF receptor signaling pathway [130]. *Hp*-OMVs, as carriers of VacA, could permit its translocation across the blood–brain barrier, eventually influencing CRF levels in the brain. This process may disrupt HPA axis homeostasis and subsequently affect bidirectional gut-brain communication.

**Fig. 4:**
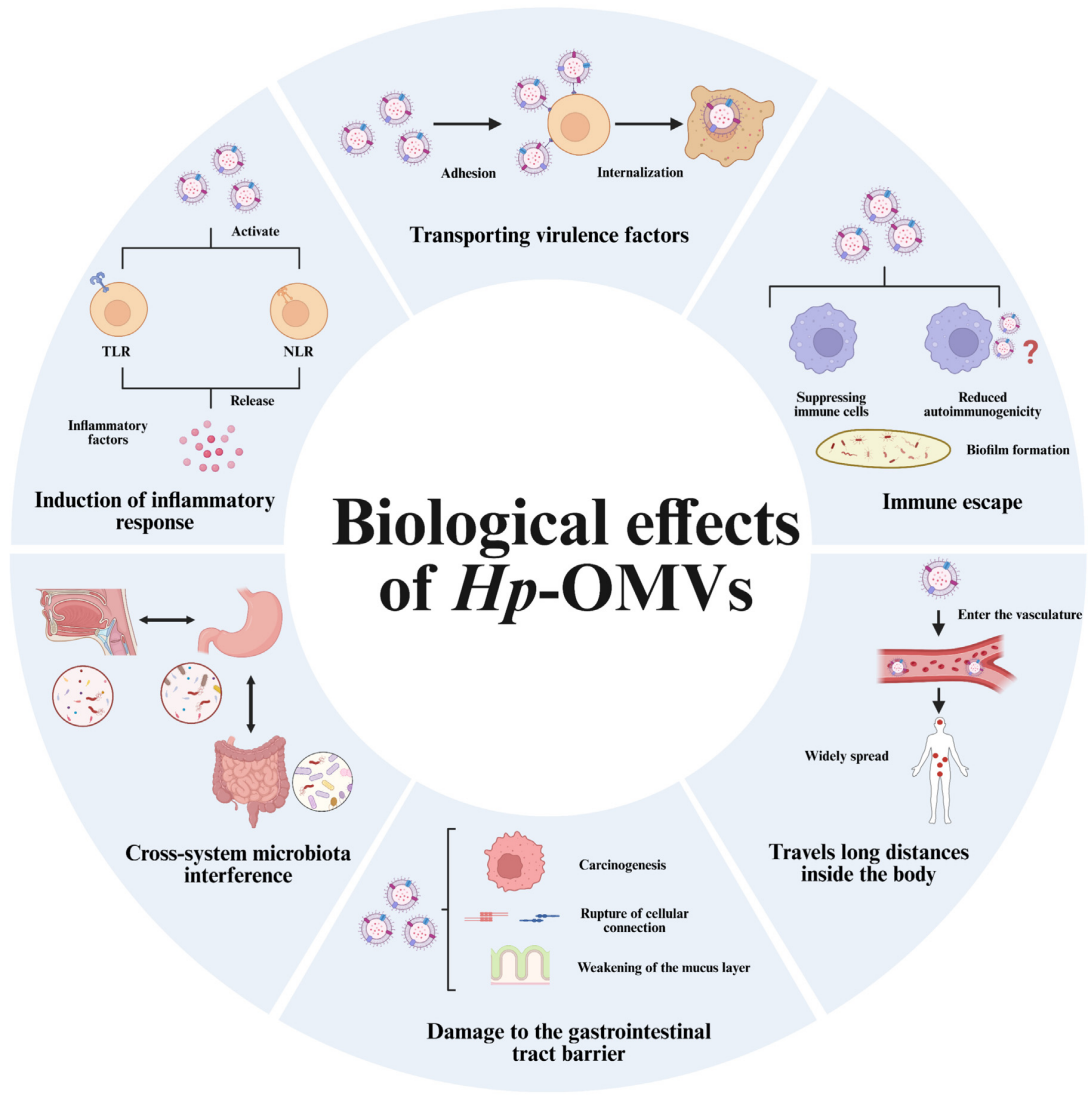
**The main functions of *Hp*-OMVs.***Hp*-OMVs contribute to the colonization and long-term survival of *Hp* in human body through the transport of virulence factors and the regulation of immune system. In particular, the multiple damage mechanisms of gastrointestinal barrier, such as carcinogenesis, cell junction destruction and mucus barrier destruction, make *Hp* show strong pathogenic ability. At the same time, the long-distance transport capability of *Hp*-OMVs also gives *Hp* the potential to produce cross-system biological effects in vivo. (Created in https://BioRender.com).

In summary, *Hp*-OMVs play a key role in a series of biological behaviors of *Hp* such as colonization and invasion. *Hp*-OMVs are a necessary condition for its adaptability and pathogenic ability (Fig. 4).

## Potential Applications of *Hp*-OMVs beyond Vaccine Development

**Fig. 5:**
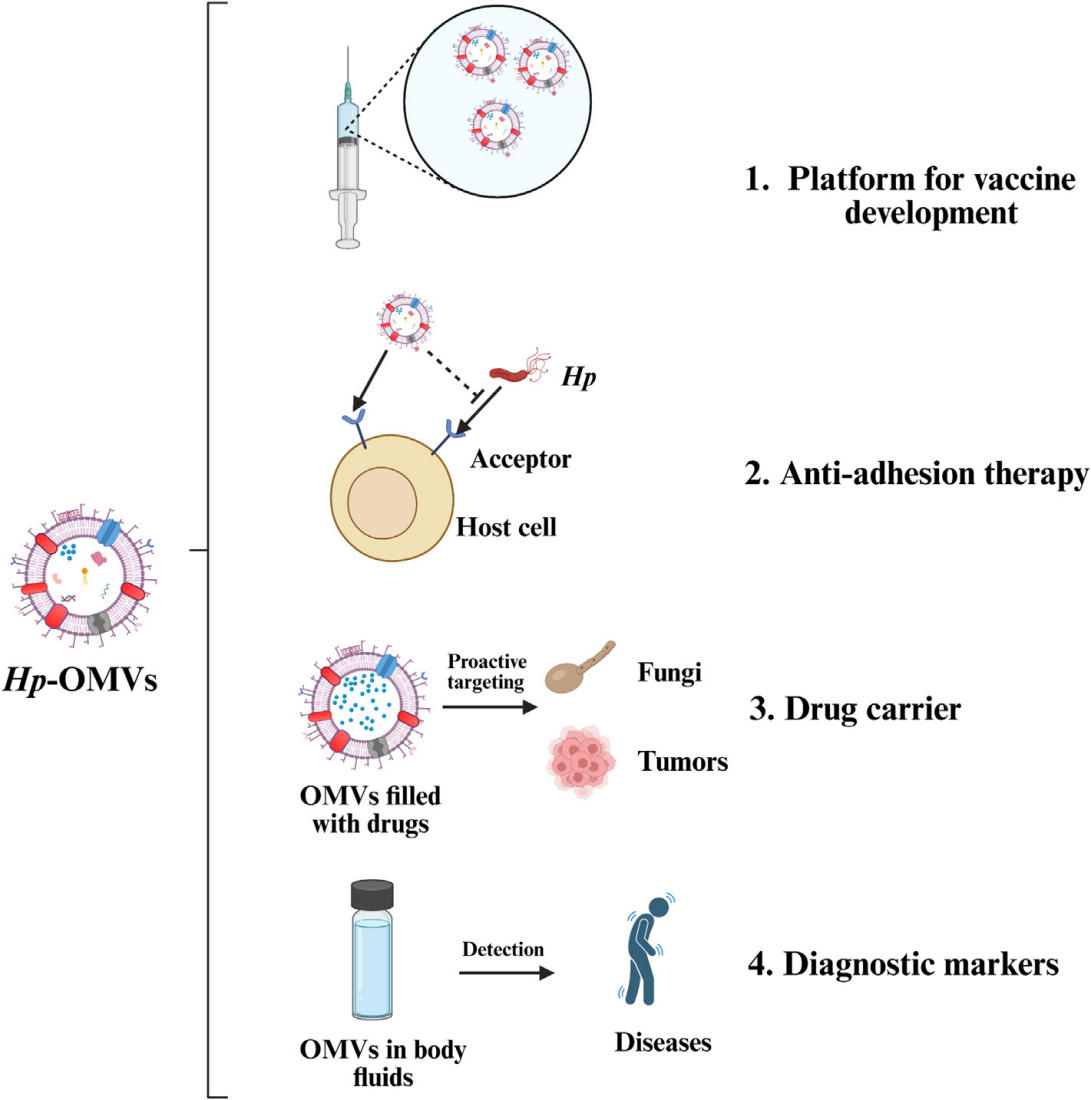
**Clinical potential of *Hp*-OMVs.***Hp*-OMVs have shown potential in a variety of clinical applications. These include platforms for vaccine development, anti-*Hp* adhesion drugs, targeted drug delivery, and biomarkers for disease diagnosis. (Created in https://BioRender.com).

*Hp* shows resistance to single and multiple drugs, as well as heterogeneous resistance, through modifications to drug targets, upregulation of efflux pump systems, and biofilm formation [131]. In recent decades, *Hp* resistance has become a major public health issue, due to rising resistance rates to clarithromycin and levofloxacin. The prevalence of primary drug-resistant *Hp* varies widely across countries in the Asia-Pacific region, necessitating urgent adjustments of *Hp* eradication strategies [132]. Consequently, we proposed that *Hp*-OMVs are being explored as a method for controlling *Hp* (Fig. 5). Prior reviews have systematically summarized the existing state of *Hp*-OMVs in vaccine research. Therefore, the follow sections will focus on analyzing their potential applications as therapeutic agents and diagnostic biomarkers [50,133].

### Anti-Adhesion Therapy

As mentioned in the first section, *Hp*-OMVs derive from the OM. Numerous adhesion-related proteins localized to the OM, such as BabA, SabA, HopZ, and OipA, have been well characterized in *Hp*-OMVs [52]. The persistence of chronic *Hp* infection in the gastric mucosa is largely primarily influenced by the interactions between its adhesion factors and the receptors on host cells. *Hp* lacks the highly specific host-cell tropism typical of viruses, however, the interaction between its virulence factors and adhesion proteins suggests a preferential affinity for specific host cell types, particularly gastrointestinal epithelial cells [134]. Therefore, utilizing the adhesive properties of *Hp*-OMVs to competitively block host cell receptors is a promising anti-adhesion therapy. A study showed that PLGA nanoparticles encapsulated with *Hp*-OMVs that lowered pathogen adhesion by competitively occupying bacterial binding sites on host cell receptors. This approach considerably decreased *Hp* adhesion by up to 50 % in a mouse model of *Hp* infection when compared to the control group, supporting the feasibility of an anti–adhesion strategy. [135].

Nevertheless, there are still several obstacles in the process from experimentation to clinical application. The safety of adhesion therapy has not been fully characterized. For instance, the prolonged adhesion of *Hp*-OMVs may lead to persistent receptor blockade, which we speculate may cause abnormal host signal transduction. Besides, *Hp*-OMVs lack large-scale production technology, and the two major issues, purity and yield, need to be addressed urgently [136].

### Drug Delivery Vehicles

OMVs possess remarkable penetration and cellular internalization capabilities, highlighting their advantages in situations where traditional drug delivery approaches fall short. Drug-loaded OMVs can penetrate deep pathological lesions with the aid of host physiological systems, guided by transport through neutrophils and macrophages. For example, low-endotoxin *E. coli* OMVs loaded with pioglitazone (PGZ) can traverse the blood–brain barrier via neutrophil-mediated inflammatory chemotaxis, accumulate in ischemic brain regions, and subsequently release PGZ to provide neuroprotective effects [137]. Similarly, Salmonella-derived OMVs loaded with doxorubicin utilize a “hitchhiking” strategy to cross the blood–brain barrier and blood-tumor barrier, offering targeted therapy while minimizing toxicity [138]. Certain bacteria secrete OMVs that contain antimicrobial substances capable of killing other bacteria or even inhibiting fungal growth. Additionally, due to the structural similarity between OMVs and OM, fusion can occur directly between strains similar to the parent bacteria, leading to the conversion of drug resistance into susceptibility [139]. Based on the fact that *Hp* is endosymbiotic with Candida albicans, which provides protection to *Hp* [114], we inferred that *Hp*-OMVs not only possess the potential to address intracellular infections, but may also represent a novel treatment for fungal infections. Additionally, nano/microrobots give an alternative strategy for targeted therapy utilizing OMVs. Nano/microrobots are devices that operate at scales from nanometers to micrometers and are acknowledged as important instruments for personalized medicine because of their high targeting specificity, precise drug delivery capabilities, and remarkable controllability [140]. OMVs loaded with anti-tumor drugs can acquire propulsion using magnetic or enzyme-driven mechanisms, ensuring targeted drug delivery to tumors. On top of that, the precision of targeting can be improved by utilizing neutrophils or macrophages as carriers [141,142]. Similarly, stimulus-responsive DDSs can react to various triggers to help with intelligent drug delivery [143].

*Hp*-OMVs master inherent targeting capabilities, making them promising candidates for targeted drug delivery. There are some core challenges we should cope with to further unveil the potential application of *Hp*-OMVs. First of all, high yield, repeatable production and purification are technically challenging, and the heterogeneity of OMVs products makes quality control more complicated. Safety is another important consideration. Although low-toxicity OMVs have been produced through genetic engineering, the residual substances such as LPS in them may still induce systemic inflammation. Although the precise targeting ability is the foundation of OMVs as a drug delivery carrier, natural OMVs may be cleared by the host's immune cells, resulting in off-target effects. Therefore, the modification of OMVs is an extra key point [144,145].

### Diagnostic Biomarkers

Extracellular vesicles (EVs) include vesicles derived from host cells as well as those originating from bacteria. The latter also called OMVs, whose clinical importance is frequently undervalued. In the context of bacterial infections, both pathogens and infected host cells release bacterial-derived EVs, referred to as OMVs. OMVs, which are enriched with bacteria-specific molecules and detectable in bodily fluids and tissues, present potential as diagnostic biomarkers [146,147]. Preliminary research has demonstrated the potential of OMVs as diagnostic biomarkers. OMVs can be conveniently isolated from bodily fluids, including plasma and urine, and subsequently analyzed using immunocapture and mass spectrometry techniques to monitor diseases like tuberculosis and pneumonia [147]. Circulating OMVs may function as potential diagnostic indicators for conditions which include liver cancer, ovarian cancer, and IBD [66]. 16S rRNA sequencing of urinary OMVs may indicate autism spectrum disorders through the detection of alterations in gut microbiota [148].

Despite those promising diagnostic potential, *Hp*-OMVs face substantial analytical and translational challenges that limit their clinical applicability. The first problem is that the similarities between OMVs and host-derived EVs (host-EVs) in terms of size, density and membrane structure make the specific identification and isolation of them difficult. It is notable that even infected host cells may release host-EVs carrying bacterial components [66,149]. Secondly, the abundance of *Hp*-OMV in body fluids is usually low, and the composition of OMVs varies among different strains and under different culture conditions, which poses extra difficulty to the detection sensitivity and quantitative accuracy [150]. More importantly, the current OMVs separation methods vary in terms of yield and purity, and various pre-analytical procedures can significantly affect the final results. These technical inconsistencies hinder the reproducibility and comparability of the test results [151]. For *Hp*-OMVs, another issue is the lack of universally accepted specific markers. A recent study has demonstrated a method for detecting and quantifying OMVs s in blood using a polymyxin B-fluorescein probe, in order to distinguish OMVs from host-EVs by targeting LPS. However, this method has a relatively small sample size and has not been validated in other types of pathogenic microorganisms [152]. Because *Hp* has a unique LPS component, we believe developing probes targeting this structure may be a promising direction.

At present, there is still a lack of solid clinical evidence. Most existing studies are small in scale and mainly serve as early-stage explorations. Many of them do not adequately control for possible influencing factors. To truly assess the diagnostic value of *Hp*-OMVs, we need large-scale, long-term studies that can help determine clear diagnostic criteria and confirm their usefulness in clinical practice. Only by overcoming these technical and biological challenges, can *Hp*-OMVs be developed into reliable, non-invasive diagnostic tools.

## Conclusion

*Hp*-OMVs selectively load various components from *Hp* and participate in several key physiological and pathogenic processes of *Hp*, which involves colonization, nutrient metabolism, information transportation and virulence factor delivery. We believe *Hp*-OMVs play the role of an important carrier of *Hp*. Furthermore, mounting evidence from research studies attests to the remarkable penetration and transport capabilities of *Hp*-OMVs. Consequently, *Hp*-OMVs have emerged as a promising research and development platform for the treatment of *Hp* and potentially other diseases.

Despite significant advancements in understanding the bioactivity of *Hp*-OMVs, several important problems remain unresolved. Recent research has identified potential associations between *Hp*-OMVs, neuronal damage, and permeability of biological barrier. Future research should utilize proteomic, metabolomic, and transcriptomic methodologies to systematically delineate signaling pathways mediated by *Hp*-OMVs, thereby elucidating their therapeutic potential. Beyond that, since *Hp*-OMVs can enter systemic circulation and can contribute to various diseases, they may operate as potential biomarkers for gut-brain axis dysfunction. Moreover, the secretion mechanism of *Hp*-OMVs has not been clarified, which may explain the current research status of no treatment targeting the secretion of *Hp*-OMVs. In conclusion, it is essential to investigate therapeutic strategies that specifically address the damage induced by *Hp*-OMVs in the context of neuroinflammation. In summary, *Hp*-OMVs constitute a promising yet insufficiently investigated element of the gut-brain axis. More research into the mechanisms mediated by *Hp*-OMVs will broaden our comprehension of *Hp* pathogenicity, thereby aiding the creation of innovative interventions for gut-brain axis disorders.

## Funding

This study was supported by National Natural Science Foundation of China [grant numbers 32,470,205 (B.R.), 82,271,033 (L.C.), 82,401,116 (Y.Z.)], Natural Science Foundation of Sichuan province [grant number 2024NSFSC0546 (B.R.)], Sichuan Science and Technology Program [grant number 2022YFS0285 (B.R.)], Health Commission of Sichuan Province [grant number chuanganyan 2024–901 (B.R.)], Develop Program, West China Hospital of Stomatology Sichuan University [grant number RD-03–202308 (L.C.)], Postdoctoral Fellowship Program of China Postdoctoral Science Foundation [grant number GZC20241122 (X.C.)] and China Postdoctoral Science Foundation [grant number 2024 M762254 (X.C.)].

## Declaration of competing interest

The authors declare that they have no known competing financial interests or personal relationships that could have appeared to influence the work reported in this paper.
